# Epitaxy from a Periodic Y–O Monolayer: Growth of Single-Crystal Hexagonal YAlO_3_ Perovskite

**DOI:** 10.3390/nano10081515

**Published:** 2020-08-02

**Authors:** Minghwei Hong, Chao-Kai Cheng, Yen-Hsun Lin, Lawrence Boyu Young, Ren-Fong Cai, Chia-Hung Hsu, Chien-Ting Wu, Jueinai Kwo

**Affiliations:** 1 Graduate Institute of Applied Physics and Department of Physics, National Taiwan University, Taipei 10617, Taiwan; f02245005@ntu.edu.tw (C.-K.C.); f01222018@ntu.edu.tw (Y.-H.L.); f03222019@ntu.edu.tw (L.B.Y.); 2 Material and Chemical Research Laboratories, Industrial Technology Research Institute, Hsinchu 31040, Taiwan; renfong@itri.org.tw; 3 National Synchrotron Radiation Research Center, Hsinchu 30076, Taiwan; 4 Taiwan Semiconductor Research Institute, Hsinchu 30078, Taiwan; 5 Department of Physics, National Tsing Hua University, Hsinchu 30013, Taiwan

**Keywords:** interfacial monolayer-induced epitaxy, atomic layer deposition, laminated multilayers, hexagonal perovskite YAlO_3_, oxide/semiconductor hetero-structure, gallium arsenide

## Abstract

The role of an atomic-layer thick periodic Y–O array in inducing the epitaxial growth of single-crystal hexagonal YAlO_3_ perovskite (H-YAP) films was studied using high-angle annular dark-field and annular bright-field scanning transmission electron microscopy in conjunction with a spherical aberration-corrected probe and in situ reflection high-energy electron diffraction. We observed the Y–O array at the interface of amorphous atomic layer deposition (ALD) sub-nano-laminated (snl) Al_2_O_3_/Y_2_O_3_ multilayers and GaAs(111)A, with the first film deposition being three cycles of ALD-Y_2_O_3_. This thin array was a seed layer for growing the H-YAP from the ALD snl multilayers with 900 °C rapid thermal annealing (RTA). The annealed film only contained H-YAP with an excellent crystallinity and an atomically sharp interface with the substrate. The initial Y–O array became the bottom layer of H-YAP, bonding with Ga, the top layer of GaAs. Using a similar ALD snl multilayer, but with the first film deposition of three ALD-Al_2_O_3_ cycles, there was no observation of a periodic atomic array at the interface. RTA of the sample to 900 °C resulted in a non-uniform film, mixing amorphous regions and island-like H-YAP domains. The results indicate that the epitaxial H-YAP was induced from the atomic-layer thick periodic Y–O array, rather than from GaAs(111)A.

## 1. Introduction

Excellent hetero-epitaxial growth, which is a material science wonder, has had strong impacts on technologies and scientific advances. Early examples are the growth of GaN/sapphire [1] and rare-earth metals/Nb/sapphire [2]. The former led to the development of the blue light-emitting diode and lasers, whilst the latter led to the discovery of long-range anti-ferromagnetic coupling through non-magnetic media [3,4], which subsequently led to the observation of a giant magnetoresistance (GMR) [5] for high-density magnetic recording. It is a challenge to perfect hetero-epitaxy, particularly when overlayers and substrates exhibit vastly different chemical bonding, e.g., metals on insulators, metals on semiconductors, insulators on semiconductors, vice versa, and many others [6,7,8,9,10,11,12,13,14,15,16,17,18,19,20,21,22].

Molecular beam epitaxy (MBE) and metal-organic chemical vapor deposition (MOCVD) have been employed to carry out most of the aforementioned epitaxial growth. Atomic layer deposition (ALD), having the advantages of self-limiting growth with an atomic accuracy and conformal coverage, has been used in industrial manufacturing, where many ALD films are amorphous oxides. ALD, nonetheless, has produced single-crystal rare-earth oxides on GaAs(001) [23,24], (111)A [23,25,26], and GaN [27]. MBE, a physical vapor deposition method, grows complex structures (including superlattices) by evaporating the constituents at the same time or sequentially. For example, MBE YBa_2_Cu_3_O_7_ single-crystalline films grew epitaxially on an SrTiO_3_ substrate by simultaneously evaporating separate sources of Y, Ba, Cu, and excited oxygen [28]. In comparison, ALD employs nano-laminated (nl) or sub-nl (snl) multilayers by depositing the constituents in sequence, followed by post-deposition annealing to form ternary-oxide epitaxial films [29,30]. ALD crystalline LaAlO_3_ films were grown on Si(001) with a buffer layer of four unit cells of MBE-SrTiO_3_ after 600 °C annealing under vacuum for 2 h [29]. Additionally, an ALD ternary single-crystal hexagonal YAlO_3_ perovskite (H-YAP) was grown on GaAs(111)A upon 900 °C rapid thermal annealing (RTA) [30]. The epitaxial growth mechanism of single-crystal perovskite by ALD approach could be different from that of poly-crystalline perovskites prepared by powder sintering [31], hydrothermal synthesis [32], and sol–gel [33] methods. Single-crystal materials prepared by ALD are thus great platforms for studying initial monolayer-induced epitaxy.

Epitaxy starts from the top surface of the substrate. The surface structure is often different from the atomic stacking in the bulk. For example, Si(100) has a 2 × 1 surface reconstruction and GaAs(100) has 4 × 6, 2 × 4, and 4 × 4 surface reconstructions. Terraces on surfaces with different step heights also affect the epi-growth. Single-crystal Gd_2_O_3_(110) and Y_2_O_3_(110) epitaxially grown on GaAs(100) have a single domain [10], while those on Si(100) have double domains rotating by 90°, induced by the respective surface reconstructions of the terraces [12]. These experimental results have shown that the surface, not the substrate bulk, determines the epitaxial growth. Similarly, a periodic configuration of adatoms on the top surface of the substrate may determine the epi-growth.

In this work, we observed a periodic array of atoms with a thickness of a monolayer or two at the interface; this was revealed in an high-angle annular dark-field (HAADF)-STEM image of the as-deposited amorphous snl ALD-Al_2_O_3_/Y_2_O_3_ multilayers on GaAs(111)A with the first film deposition of three cycles of ALD-Y_2_O_3_. The initial ALD-Y_2_O_3_ growth resulted in faint broad reflection high-energy electron diffraction (RHEED) streaks superimposed with the sharp ones from the underlying substrate. The Y–O monolayer, not GaAs, induced the epi-growth of H-YAP by annealing the amorphous snl ALD multilayers. This, however, was not achievable when using other snl ALD-Y_2_O_3_/-Al_2_O_3_ multilayers on GaAs(111)A, but employing the first deposition of three cycles of ALD-Al_2_O_3_.

## 2. Materials and Methods

**Figure 1:**
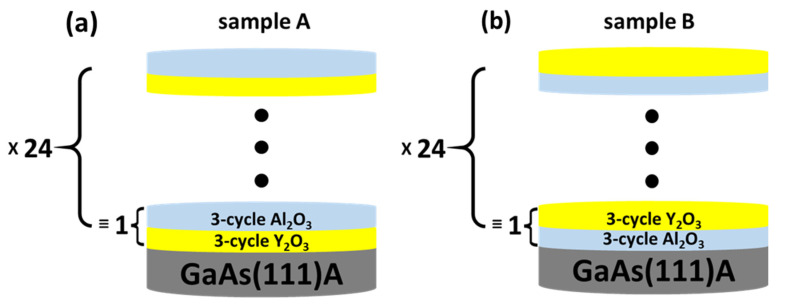
Schematics of snl multilayers of 24 periods of (**a**) atomic layer deposition (ALD)-Al_2_O_3_/-Y_2_O_3_ (sample A) and (**b**) ALD-Y_2_O_3_/-Al_2_O_3_ (sample B), with each constituent consisting of 3 ALD cycles.

The samples were prepared in a growth/analysis ultra-high vacuum (UHV) multi-chamber system (Designed and constructed by M. Hong, J. Kwo, and the group members, Taiwan, with chambers/parts from various countries.) [34,35]. All of the chambers were connected through UHV modules under ~10^−10^ torr to ensure intactness of the pristine surfaces and interfaces during the sample transfers. Epitaxial GaAs layers were grown on GaAs(111)A in the solid-source GaAs-based MBE chamber. The epi-wafers were transferred under UHV to the ALD reactor for the deposition of the snl multilayers, which included 24 periods of ALD-Al_2_O_3_ (three cycles)/-Y_2_O_3_ (three cycles) [30]. Y(EtCp)_3_/deionized H_2_O and TMA/deionized H_2_O were used as co-reactants for constituents of Y_2_O_3_ and Al_2_O_3_, respectively. Y(EtCp)_3_ denotes tris(ethylcyclopentadienyl) yttrium and TMA denotes trimethylaluminum. Two samples of different stacking orders were prepared. Figure 1 shows the schematics of sample A and B, where A has the ALD-Y_2_O_3_ (three cycles) as the first layer and B has the ALD-Al_2_O_3_ (three cycles) as the first layer. We used in situ RHEED to monitor the sample growth. For comparison, we studied the growth of pure ALD-Y_2_O_3_ and -Al_2_O_3_ films from the initial stage to a nm thickness.

After the ALD, all of the samples were taken out from the UHV system and annealed to 900 °C in a helium atmosphere for 30-60 s using RTA. We characterized the crystalline structure of the samples using synchrotron radiation X-ray diffraction (SR-XRD) (Huber, Rimsting, Germany) at the National Synchrotron Radiation Research Center (NSRRC). We studied the detailed atomic packing by high-resolution high-angle annular dark-field (HAADF) and annular bright-field (ABF) spherical aberration (Cs)-STEM located at National Taiwan University (NTU) and the Industrial Technology Research Institute (ITRI). The STEM experiments were performed on an aberration-corrected (0.9 Å probe size) JEOL 2100F scanning transmission electron microscope (JEOL, Tokyo, Japan), operated at an accelerating voltage of 200 kV at NTU, and spherical aberration (Cs) corrected STEM (JEOL, JEM-ARM200F, Tokyo, Japan), operated at an accelerating voltage of 200 kV, at the Industrial Technology Research Institute (ITRI). We prepared the STEM samples using mechanical polishing and a focused ion beam (FIB) at the Taiwan Semiconductor Research Institute (TSRI).

## 3. Results and Discussion

**Figure 2:**
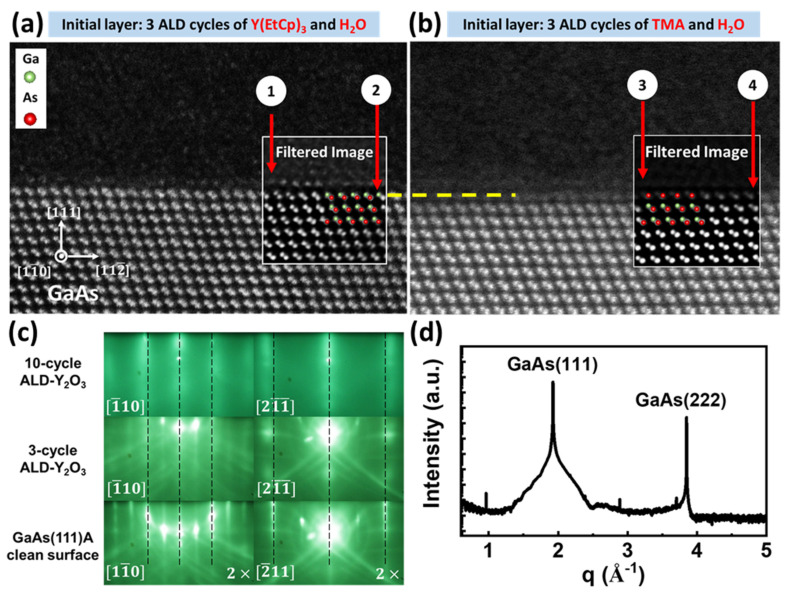
Cross-sectional HAADF-STEM images of (**a**) sample A and (**b**) sample B, as viewed along GaAs $\left[1\bar{1}0\right]$ zone axis, (**c**) RHEED patterns of epi GaAs(111)A, 3-cycle ALD-Y_2_O_3_, and 10-cycle ALD-Y_2_O_3_, and (**d**) SR-XRD specular scan of (**a**) sample A. A dashed yellow line marks the top GaAs layer and a model of GaAs stacking in green and red is overlaid in Figure 2a,b. Arrow (1) points out the ordered Y adatoms. Arrow (2) points out the GaAs(111)A surface with Ga on top. Arrow (3) points out that there is no observation of ordered Al or Y adatoms. Arrow (4) points out the top GaAs dumbbells with visible As.

The high-resolution HAADF-STEM image (Figure 2a) of sample A (Figure 1a) in the as-deposited condition shows an amorphous ALD snl layer on crystalline GaAs(111)A. No diffraction peaks except for the GaAs(111) and (222) reflections were found in the SR-XRD specular scan, shown in Figure 2d, which confirmed the lack of a long-range order of the deposited film along the surface normal. The Ga–As dumbbell pairs in the GaAs(111)A epilayer are clearly resolved in the STEM image and the corresponding crystalline orientation is labeled in the figure. Note that the atomic stacking is always terminated with Ga in GaAs(111)A substrate and with the epitaxial growth. We studied the surface electronic structure of the epi-GaAs(111)A using in situ synchrotron radiation photoelectron spectroscopy (SRPES), confirming the Ga termination [36].

It is noteworthy that a periodic array of a single atomic layer or two is visible at the hetero-interface above the topmost layer of GaAs dumbbells. The periodic array is very likely a Y–O atomic template from the first three ALD cycles using Y-precursor Y(EtCp)_3_ and water. The Y–O bonded with Ga, forming Y–O–Ga. The in-plane symmetry of GaAs(111)A, a thin Y–O adatom array, and thicker Y_2_O_3_ was studied using in situ RHEED, and will be discussed later.

In comparison, the high-resolution HAADF-STEM image of sample B in the as-deposited condition (Figure 2b) shows an amorphous ALD snl layer on the crystalline GaAs(111)A similar to that in sample A; however, no periodic array of adatoms on top of GaAs(111)A was observed, different from what was observed in sample A. Note that sample B in Figure 1b has 24 periods of ALD-Y_2_O_3_ and -Al_2_O_3_ in an snl structure, similar to sample A, but with the initial layer being three cycles of ALD-Al_2_O_3_. The Ga–As dumbbells in the GaAs(111)A epilayer are clearly resolved and remain intact from the bulk to the top surface of the substrate in sample A in both unfiltered and filtered images (Figure 2a). In sample B, the Ga–As dumbbells in GaAs(111)A are also clearly resolved; however, the very top dumbbells are blurred in the unfiltered image. The filtered image revealed the existence of As, which was bonded with some atoms, whose contrast is not as clear as that of Ga in sample A. See and compare the unfiltered and filtered images in Figure 2a,b. The TMA and H_2_O of the three ALD cycles in sample B could interact with the top Ga, resulting in a less ordered interface structure.

The in situ RHEED patterns upon the initial growth of ALD Y_2_O_3_ on the GaAs(111)A revealed ordered and crystalline Y_2_O_3_ from 3 to 10 cycles, similar to our earlier work reported in Ref. [23]. The bottom panels of Figure 2c show sharp, streaky reconstructed RHEED patterns of the clean epi-GaAs(111)A-(2 × 2) with Kikuchi arcs. With 3-cycle ALD-Y_2_O_3_ deposition (middle panels), faint broad streaks, superimposed with the underlying GaAs pattern, were observed. After 10 cycles (top panels), the broad streaks became dominant, which manifested the crystalline nature of the thicker Y_2_O_3_ layer. The RHEED k-spacing in the thin ALD oxide is slightly larger than that of epi-GaAs, marked by dashed lines. The trend continues with 10-cycle ALD-Y_2_O_3_, where no Kikuchi arcs were observed.

Note that three to five cycles of ALD-Y_2_O_3_ as the initial deposition are employed to give a complete coverage, namely 1 monolayer, on GaAs surface, which may not be attained with two cycles of ALD-Y_2_O_3_ [37]. The more cycles of ALD-Y_2_O_3_ or ALD-Al_2_O_3_ layer may cause less mixing uniformity. Therefore, three cycles of ALD-Y_2_O_3_ and ALD-Al_2_O_3_ are suitable for the purpose of homogenous chemical composition and attaining the monolayer limit at the initial growth stage. The k-spacing in the RHEED patterns increases from GaAs(111)A, from 3-cycle, to 10-cycle, to 20-cycle (not shown) ALD-Y_2_O_3_, consistent with the corresponding decrease in the in-plane r-spacing of the observed HAADF-STEM images of GaAs(111)A, the 3-cycle ALD-Y_2_O_3_ (Figure 2a), and thicker Y_2_O_3_ (not shown). The k-spacing of 3-cycle ALD-Y_2_O_3_ observed from RHEED is 1.03 times larger than that of the GaAs substrate, which corresponds to the d-spacing of 0.115/1.03 = 0.112 nm. The Y–Y distance of 3-cycle ALD-Y_2_O_3_ observed from HAADF-STEM (Figure 2a) is approximately 0.343 nm, which is ~1.01 times smaller than the Ga–Ga distance of 0.346 nm. These data show the consistency between the measured results of 3-cycle ALD-Y_2_O_3_ using RHEED and HAADF-STEM. Note that 0.115 nm is the in-plane d-spacing of GaAs, as determined from STEM and SR-XRD.

Studies of a 5 nm thick single-crystal Y_2_O_3_ on GaAs(111)A using HAADF-STEM showed the in-plane Y–Y distance of 0.326 nm along the Y_2_O_3_ [$\bar{1}\bar{1}2]$ direction, indicating that the corresponding d-spacing of Y_2_O_3_($8\bar{4}\bar{4}$) is 0.326/3 = 0.109 nm. The measured SR-XRD off-normal diffraction peaks gave the in-plane d-spacing of Y_2_O_3_($8\bar{4}\bar{4}$) of 0.108 nm. The k-spacing observed from the RHEED pattern of the 10-cycle ALD-Y_2_O_3_ is 1.08 times larger than that of the GaAs substrate, indicating that the d-spacing is 0.115/1.08 = 0.106 nm. The measured values obtained using HAADF-STEM, RHEED, and SR-XRD upon the initial deposition of a monolayer to thicker films are consistent with each other.

Different from the RHEED studies of ALD-Y_2_O_3_ on GaAs(111)A, as discussed above, those of ALD-Al_2_O_3_ on GaAs(111)A, from the initial ALD oxide growth to thicker films, are non-crystalline (not shown). Our earlier work indicated that 10 cycles of ALD-Al_2_O_3_ were not able to passivate 100% of a GaAs(001)-4 × 6 surface [38]. Therefore, the initial three cycles of ALD-Al_2_O_3_ on sample B would leave bare (not passivated) patches of GaAs(111)A for the subsequent three cycles of ALD-Y_2_O_3_ to bond with, resulting in mixed coverages of Al_2_O_3_ and Y_2_O_3_ on top of GaAs(111)A on sample B. The RHEED observations on the initial growth of ALD-Y_2_O_3_ and ALD-Al_2_O_3_ on GaAs(111)A are consistent with the HAADF-STEM images near the interface region of samples A (Figure 2a) and B (Figure 2b). The as-deposited condition of sample A showed a periodic adatom array, whereas that of sample B revealed no visible periodic adatom array. Moreover, the surface Ga–As dumbbells interacted with the 3-cycle of ALD-Al_2_O_3_ differently from those with the 3-cycle of ALD-Y_2_O_3_ in the as-deposited sample A, whose top GaAs dumbbells remained intact upon 3-cycle ALD-Y_2_O_3_ deposition. The HAADF-STEM images show amorphous ALD snl multilayered thin films for both samples A and B in the as-deposited condition.

Previously, we studied the initial chemical bonding of the first half- and one-cycle ALD-Y_2_O_3_ on GaAs(001)-4 × 6 on an atomic scale using in situ SRPES [39]. Y(EtCp)_3_ precursors reside on the faulted As atoms and undergo a charge transfer to the bonded As atoms. The next ALD half-cycle of H_2_O molecules removes the bonded As atoms, and the oxygen atoms bond with the Ga atoms underneath, forming Y–O–Ga bonding at the interface. Y–O–Ga prevented the interdiffusion between Y_2_O_3_ and GaAs upon RTA to high temperatures, regardless of ALD- or MBE-Y_2_O_3_, as evidenced from the attainment of low D_it_ and a low electrical leakage current in the Y_2_O_3_/GaAs(001)-4 × 6 [40,41]. Note that the top surface atoms of GaAs(111)A are Ga [36,42], while those of (001)-4 × 6 are As [38,43]. Therefore, forming Y–O–Ga bonding is easier for ALD-Y_2_O_3_ on GaAs(111)A than that on GaAs(001).

**Figure 3:**
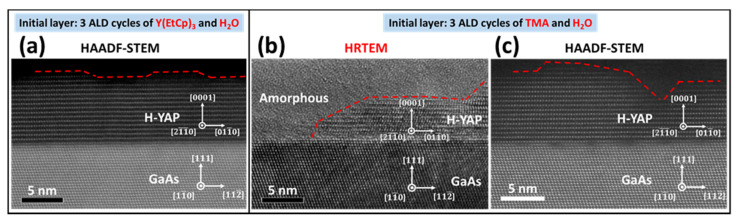
(**a**) Cross-sectional HAADF-STEM image of sample A after 900 °C annealing, showing a uniform single-crystal single-domain H-YAP across GaAs(111)A with an atomically smooth interface, while (**b**) is a cross-sectional HRTEM image of sample B after 900 °C annealing, showing a non-uniform film consisting of regions of single-crystal H-YAP and amorphous area. Figure 3 (**c**) is a cross-sectional HAADF-STEM image of sample B after 900 °C annealing, showing island-like H-YAP domains. HAADF-STEM images were viewed along the H-YAP [$2\bar{1}\bar{1}0$] zone axis, which is also the GaAs [$1\bar{1}0$] zone axis.

**Figure 4:**
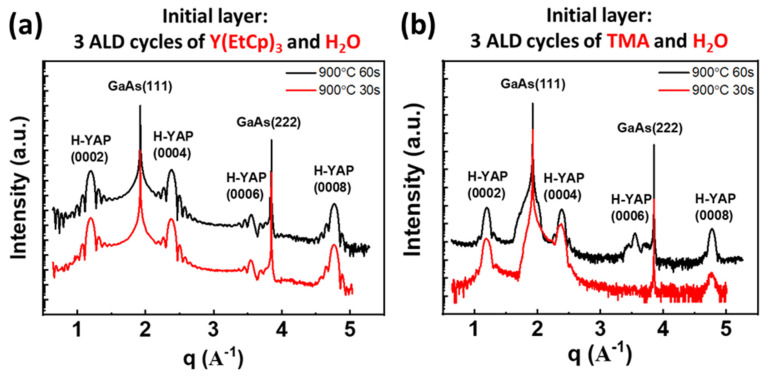
XRD radial scans along the surface normal of sample A (**a**) and sample B (**b**), both with 900 °C rapid thermal annealing (RTA) for 30 and 60 s.

RTA to 900 °C transformed the amorphous snl ALD multilayer in sample A to single-crystal single-domain H-YAP, as shown in an HAADF-STEM image in Figure 3a. The hetero-interface was atomically ordered and morphologically smooth and sharp. The corresponding SR-XRD normal scans are shown in Figure 4a.

In comparison, the structure of sample B after 900 °C RTA was not uniform. Island-like H-YAP crystalline grains formed on parts of the substrate, while the rest were amorphous, as shown in a high-resolution transmission electron microscopy (HRTEM) image (Figure 3b). The interface of the annealed sample B is not as smooth as that of the annealed sample A, particularly in the amorphous region. The HAADF-STEM image of a crystalline H-YAP region in the annealed sample B is shown in Figure 3c, which reveals an atomic-scale microstructure of a similar quality to that of sample A. Nonetheless, the H-YAP region exhibits an island-like morphology, similar to what is exhibited in Figure 3b.

The XRD radial scans along the surface normal of samples A and B after RTA to 900 °C for 30 and 60 s are illustrated in Figure 4a,b. The abscissa denotes the scattering vector, q, whose magnitude is 4π× sin(2θ/2)/λ, where 2θ and λ are the scattering angle and X-ray wavelength, respectively. Apart from the intense GaAs(111) and (222) Bragg peaks, four additional diffraction peaks were observed, where the peak locations agree well with the expected positions of the H-YAP(0002), (0004), (0006), and (0008) reflections, verifying the c-axis-oriented H-YAP. Moreover, the extensive persistence of Pendellösung fringes near the H-YAP reflections in sample A provides additional evidence of its sharper interfaces and better crystallinity compared with those in sample B. These XRD observations are consistent with the STEM results.

**Figure 5:**
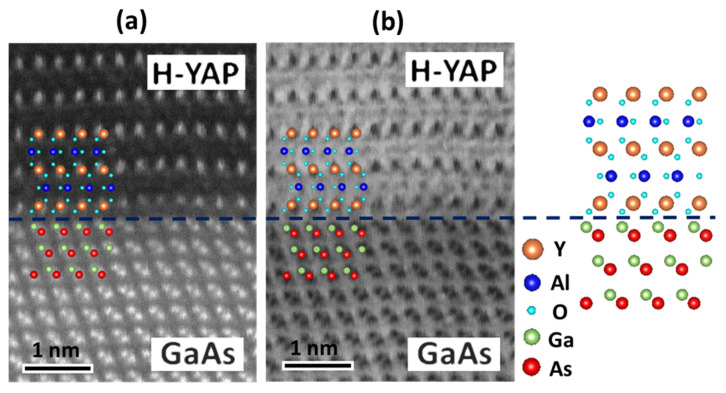
Cross-sectional (**a**) HAADF- and (**b**) ABF-STEM image of enlarged area of H-YAP/GaAs interface of annealed sample A. A dash line indicates the interface between top GaAs and H-YAP. Models of H-YAP and GaAs overlay with the constituents in Figure 5a,b. HAADF- and ABF-STEM images of H-YAP were viewed along [$2\bar{1}\bar{1}0$] zone axis and those of GaAs were viewed along [$1\bar{1}0$] zone axis.

The high-resolution HAADF- and ABF-STEM images (Figure 5a,b) provide valuable information about the bonding at the interface and inside the H-YAP. The images are identical for the whole film, which is H-YAP in the annealed sample A and the H-YAP domains in sample B. We overlaid the atomic models of H-YAP and GaAs on both figures. The good match between the models and the imaged atomic columns indicates that the constituent atoms were located at the expected positions, having the expected bonding length and angle. We observed the Ga–As pairs and the Y atoms periodically located in the hexagonal YAP lattices. The observed interplanar distances between neighboring Y atomic planes along both lateral [01$\bar{1}$0] and normal [0001] directions are well matched with those in the atomic models, which were drawn using the VESTA software [44]. In the HAADF image, the intensity scattered by an atom scales with the atomic number Z as Z^1.7^ [45]. Therefore, the HAADF-STEM image contrast is dominated by heavier Y, Ga, and As atoms; the light Al and O atoms, which are situated between Y atoms, are barely observable in Figure 5a.

In the ABF-STEM images, with black atom contrast, the heavier atoms correspond to dots with a larger size and darker colors. Although ABF-STEM images exhibit smaller image contrast than the HAADF-STEM ones, both light and heavy atomic columns are visible simultaneously [46]. The ABF-STEM image thus shows not only the Ga–As pairs of the epi-GaAs and the Y of H-YAP films, but also, critically, the Al and O sites. The enlarged ABF-STEM image of an interface region of annealed sample A is shown in Figure 5b, in which the largest and darkest dots are the heaviest Y atoms. Between the Y layers stacked along the H-YAP c-axis, the gray and smaller dots reveal the locations of the Al atom and match those in the atomic models, as depicted in Figure 5a,b.

The oxygen atoms have the smallest size and are the most difficult to observe among all the elements in this work. The dark features extended from the nearby metal atoms in H-YAP can be identified as oxygen atoms, as indicated by the red dots in Figure 5b. Furthermore, the clouded region between the H-YAP film and GaAs substrate indicates the existence of O atoms at the interface between Y and Ga atoms. We have elucidated the interfacial atomic arrangements, namely only O atoms being between Y and Ga atoms. The observations indicate that the interfacial bonding between H-YAP and GaAs(111)A is Y–O–Ga, which comes from the initial single atomic layer of periodic Y–O. The sharp interface also indicated that Y–O–Ga bonding is very strong and stable and sustains 900 °C RTA without detectable deterioration.

## 4. Conclusions

The substrate surface initiates and determines the epi-growth, which involves deposited films and the substrates underneath. It is rare to see epi-growth on a periodic array of adatoms, which is not part of the native substrate surface. In this work, we utilized atomic layer deposition (ALD), which is a self-limiting growth method with an atomic accuracy and conformal coverage, to tailor the configuration of the adatoms on the GaAs(111)A surface. The first film deposition of three cycles of ALD-Y_2_O_3_ gave a periodic array of atoms with a thickness of a monolayer or two at the interface; this was observed using careful studies of HAADF- and ABF-STEM, in situ RHEED, and our previous work of the in situ synchrotron radiation photoemission. The Y–O monolayer, not GaAs, induced the epi-growth of single-crystal hexagonal yttrium aluminum perovskite (H-YAP) by annealing the amorphous sub-nano-laminated (snl) ALD-Al_2_O_3_/-Y_2_O_3_ multilayers. The single-crystal H-YAP was uniform over the STEM-studied area in the GaAs wafer, and had an atomically sharp interface with the substrate. The persistence of the Pendellösung fringes of X-ray scattering radial scans along the surface normal near the H-YAP reflections over a large q range indicate sharp interfaces and excellent crystalline structures. In comparison, the amorphous snl ALD-Y_2_O_3_/-Al_2_O_3_ multilayer on GaAs(111)A with the initial three ALD cycles of Al_2_O_3_ resulted in an annealed film consisting of amorphous regions and island-like single-crystal H-YAP grains.

With the same substrate of GaAs(111)A, the initial film depositions of 3 ALD cycles of Y_2_O_3_ or Al_2_O_3_ produced films of entirely epitaxial single-crystal H-YAP with an atomically smooth interface or mixed amorphous/single-crystal regions, respectively. It is the periodic Y–O adatom array, rather than the GaAs substrate, which induced the epi-growth of single-crystal H-YAP. Single-crystal H-YAP was successfully grown on GaN, and is expected to be grown on Si using the method discussed here. Our results may lead to novel epitaxial growth by tailoring the substrate surfaces using a foreign atomic-layer thick periodic adatom array for producing desirable phases, opening up a new chapter in hetero-epitaxy.
